# Heating Has No Effect on the Net Protein Utilisation from Egg Whites in Rats

**DOI:** 10.1155/2017/6817196

**Published:** 2017-02-26

**Authors:** Ryosuke Matsuoka, Yayoi Takahashi, Mamoru Kimura, Yasunobu Masuda, Masaaki Kunou

**Affiliations:** R&D Division, Kewpie Corporation, 2-5-7 Sengawa Kewport, Sengawa-Cho, Chofu-Shi, Tokyo 182-0002, Japan

## Abstract

Egg whites (EW) are a good source of protein; however, they are typically heated prior to consumption. Therefore, we investigated the effects of different heating conditions on the protein utilisation rate of EW. Male Sprague-Dawley rats (*n* = 36, 198 ± 1 g) were divided into six groups and fed American Institute of Nutrition-76 chow containing unheated EW, soft-boiled EW, boiled EW, milk whey protein, soybean protein, or no protein over a 10-day period using pair-feeding. Urine and faeces were sampled daily beginning on day 5 to measure nitrogen content and the net protein utilisation (NPU) rate. The soybean protein group had a significantly lower level of food intake and was thus excluded from subsequent analyses. The NPU value was similar among the unheated, soft-boiled, and boiled EW groups (97.5 ± 0.4, 96.5 ± 0.1, and 96.5 ± 0.7, resp.). The EW group values were significantly higher than the whey group values (90.5 ± 1.0). These results show that EW serve as a good source of protein, irrespective of heating.

## 1. Introduction

Chicken eggs are highly nutritious and contain most recommended nutrients, except vitamin C and dietary fibre. The egg yolk contains many important nutrients, including proteins, lipids, vitamins, and minerals, whereas egg whites (EW) principally contain proteins, water, and almost no fat [1]. Extensive research has been conducted on egg yolks, specifically on the health benefits of egg yolk phospholipids. Egg yolk phospholipids lower the cholesterol level and improve fatty liver and brain function [2–4]. With regard to egg white protein on the other hand, many reports have described allergies [5, 6], while, in terms of health function, the iron absorption enhancing effect [7] and cholesterol-lowering effect [8, 9] have been reported.

EW are a good source of protein, as evidenced by their amino acid score of 100 and high rate of net protein utilisation (NPU) [1, 10]. A previous study reported that unheated EW contain protease inhibitors, which can inhibit the activity of trypsin, elastase and chymotrypsin [11]. However, EW increased trypsin and chymotrypsin activity to a greater degree than egg yolks or whole eggs; thus, the function of protease inhibitors in EW is not fully understood [12]. Heated EW are thought to inhibit protease inhibitor activity and increase protein absorption. However, it has been reported that chymotrypsin activity is inhibited to a greater degree in heated whole eggs, suggesting that protein absorption and NPU are greater in unheated eggs [12]. Because proteins are modified by heat treatment, different heat conditions may result in distinct NPU changes. However, studies on heat treatments and protein absorption have typically used dried EW [10] without specifying if the EW were unheated or heated. Therefore, the effects of heating remain unclear, even though most EW are ingested after heating. Here, we investigate the effect of different heating conditions on the NPU of EW and compare these data with the widely used sources of protein: milk whey and soy.

## 2. Materials and Methods

### 2.1. Materials

We used freeze-dried, powdered, and nonsterilized EW (Kewpie Egg Corporation, Tokyo, Japan) in three forms: unheated (unheated EW), heated at 65°C for 5 min (soft-boiled EW), and heated at 95°C for 10 min (boiled EW). Milk whey protein was purchased from Arla Foods Japan and Arla Foods Ingredients, Japan K.K. (Tokyo). Soy protein was purchased from Fuji Protein Technologies Inc. (Osaka). The protein content of each sample was determined by analysing the nitrogen content with the Dumas method [13] and multiplying by a factor of 6.25. The protein content was 88.8 g/100 g for unheated EW, 91.6 g/100 g for soft-boiled EW, 86.2 g/100 g for boiled EW, 86.6 g/100 g for milk whey protein, and 92.7 g/100 g for soy protein.

### 2.2. Animals and Diets

Male Sprague-Dawley rats (198 ± 1 g, *n* = 36) were kept in metabolic cages (Toyoriko Co. Ltd., Tokyo) under a 12-h light cycle (8:00 to 20:00) at a temperature of 23 ± 1°C and humidity of 50 ± 2%.

The test diet was prepared with American Institute of Nutrition- (AIN-) 76 chow [14] and 10% or 0% protein (unheated EW, soft-boiled EW, boiled EW, milk whey protein, or soy protein), 15% beta-corn starch, 5% cellulose, 3.5% mineral mix (AIN-76), 1% vitamin mix (AIN-76), 5% corn oil, and 0.2% choline bitartrate and sucrose. The control group was fed 0% protein to calculate the level of metabolic nitrogen excretion in the faeces and urine (32.4 mg/5 days and 125 mg/5 days, resp.). We used AIN-76 chow and a 10% protein diet because the protein utilisation rate is easier to measure at lower protein levels. This approach was previously described [15, 16], and thus, facilitates the comparison of our results with those of other studies. The rats were fed this diet over a 10-day period using pair-feeding with free access to distilled water for drinking. Faeces and urine were collected for the last 5 days of feeding immediately before the completion of the experiment.

This experiment was conducted under the guidelines for Animal Experiments, Law number 105 and Notification number 6 of the Government of Japan.

### 2.3. Chemical Analysis

The protein content of faeces and urine was calculated by measuring the nitrogen content with the Dumas method [13] and then multiplying it by a protein conversion factor (6.25). The protein efficiency rate, digestibility (also known as the absorption rate), and NPU were calculated using the following equations [17]:

(1) Protein  efficiency=body  weight  gainprotein  intake,Digestibility=N  intake  faecal  N−metabolic  faecal  NN  intake×100,NPU=Digestibility−N  intake−urine  N−metabolic  urine  NN  intake×100.

### 2.4. Statistical Analysis

All data are presented as the mean ± standard error. Statistical analyses were performed using a one-way analysis of variance test followed by Tukey's test. *p* values < 0.05 were considered significant. The analysis was completed using SPSS II for Windows (SPSS Inc., Tokyo, Japan).

## 3. Results

### 3.1. Growth Variables

The soy group had a significantly lower level of food intake (17.0 ± 0.52 g/days) when compared with the other groups. Therefore, this group was excluded from all statistical analyses. There were no significant differences among the four treatment groups in terms of body weight gain, amount of food intake and amount of protein intake (Table 1). The feed efficiency and protein efficiency rates were similar among the unheated, soft-boiled and boiled EW groups. All EW groups showed significantly higher efficiency values when compared with the whey group.

### 3.2. Nitrogen Content of Faeces and Urine

There were no significant differences in the amount of urine and faeces produced among the four treatment groups (Table 2). The unheated, soft-boiled, and boiled EW groups had similar nitrogen contents in their faeces and urine, which were all significantly lower than those of the whey group.

### 3.3. Protein Efficiency, Digestibility and NPU

**Figure 1:**
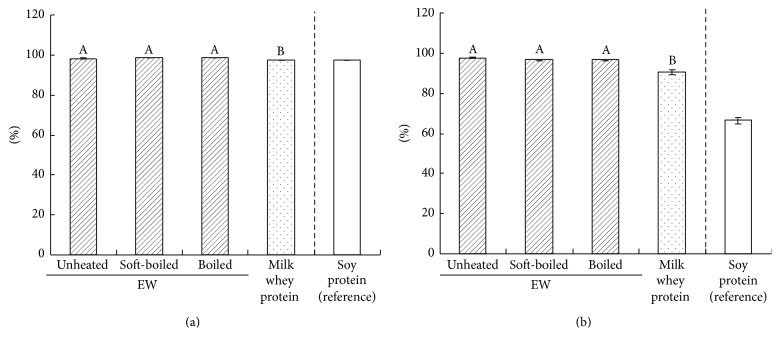
Digestibility (a) and net protein utilisation (b) of heat-treated egg white (EW) in rats. Mean ± standard error (SE) of six rats. Superscript letters indicate a significant difference (Tukey's test, *p* < 0.05).

The digestibility and NPU were similar among the unheated, soft-boiled, and boiled EW groups. The EW group values were significantly higher than those of the whey group (*p* < 0.05) (Figure 1).

## 4. Discussion

We found that the NPU for EW was approximately 97%, irrespective of the heating. Furthermore, the NPU for EW was significantly higher than that for milk whey protein (90%). This result was comparable to previously reported values [10, 18].

Although unheated EW contain protease inhibitors, soft-boiled and boiled EW have similar NPU values. Previous reports showed that the trypsin inhibitor ovomucoid is thermally stable [19]. However, ovomucoid is broken down by pepsin in the stomach in both its unheated and heated states [20]; thus, its trypsin-inhibiting effects may not occur in the gastrointestinal tract. In contrast, ovalbumin, the main protein constituent of EW, is readily broken down by pepsin in its heated state [20], suggesting that heated EW have higher digestibility and NPU. However, in the present study, we found that the digestibility and NPU of heated EW were comparable to those of unheated EW, possibly because the intake of unheated EW increases protease activity [12].

The protein in EW has the same amino acid score as milk whey protein and soy protein (i.e., 100); therefore, these proteins have comparable digestibility (Figure 1(a)). However, EW have higher NPU than both milk whey protein and soy protein (Figure 1(b)). Indeed, EW contain the same amount of branch-chain amino acids as milk whey protein but a higher content of sulphur-containing amino acids (cysteine and methionine) [21].

These findings indicate that EW can be used in nutritional supplements to maintain physical strength in elderly individuals (e.g., in a liquid diet) or to help build muscle more effectively during exercise. In fact, a combination of exercise and EW protein increases muscle mass and muscular strength [22]. Our present study measured the NPU in rats. However, previous studies on humans have used whole eggs and showed that the NPU is affected by calorie and nitrogen intake. Therefore, the NPU should be considered in terms of the overall meal [23].

Because the rate of protein utilisation from EW is high in the body, EW are a useful protein source for children. However, this type of protein contains allergenic substances. There is considerable anticipation regarding the processing of EW to remove allergenic substances and break down the proteins into peptides via enzymes [24, 25]. The NPU values of egg yolk proteins and egg yolk peptides are comparable [26]; thus, this is also likely to be true of the proteins and peptides from EW. This prediction remains to be confirmed.

EW also contain avidin. Avidin complexes with biotin to impair its digestion [27], which can lead to a biotin deficiency if consumed in large amounts that exceed the capacity of pepsin [20, 28]. Intestinal bacteria also create biotin [29]; therefore, the combination of various factors, including the state of intestinal bacteria, can explain biotin deficiency. However, this effect can be counteracted by the concurrent eating of foods containing both biotin and EW.

Despite pair-feeding, rats in the soy group exhibited a significantly lower food intake than the other protein groups investigated. Soy protein contains less methionine than casein, and rats (particularly growing rats, as used in the present study) that are fed a methionine-imbalanced diet exhibit reduced food intake [30, 31]. Furthermore, soy protein promotes the secretion of cholecystokinin, a gastrointestinal hormone that reduces appetite [32]. A previous study showed that consumption of an egg for breakfast rather than a bagel provided a sense of satiation [33]. However, in the present study, the rats did not reduce their food intake after eating EW (Table 2). Thus, it appears that the mechanism that provides a sense of satiation after the consumption of an egg is different from that elicited by soy protein. We found that unheated, soft-boiled, and boiled EW had similar NPU values, which were significantly higher than that of milk whey protein. Our findings indicate that EW remain a good source of protein, irrespective of heating.

## Figures and Tables

**Table 1:** Growth variables in rats fed a diet containing egg whites (EW) (unheated, soft-boiled, or boiled), milk whey protein, or soy protein.

	Unheated EW	Soft-boiled EW	Boiled EW	Milk whey protein	Soy protein (reference)
Initial body weight (g)	199 ± 2	198 ± 3	198 ± 3	198 ± 3	198 ± 3
Final body weight (g)	252 ± 4	254 ± 2	255 ± 4	245 ± 3	215 ± 4
Body weight gain (g/day)	5.33 ± 0.19	5.55 ± 0.12	5.68 ± 0.14	4.71 ± 0.15	1.71 ± 0.25
Food consumption (g/day)	18.0 ± 0.2	18.3 ± 0.2	18.4 ± 0.1	18.5 ± 0.4	17.0 ± 0.5
Food efficiency^*∗*^	0.294 ± 0.008^a^	0.303 ± 0.009^a^	0.308 ± 0.007^a^	0.255 ± 0.011^b^	0.100 ± 0.012
Protein consumption (g/day)	1.41 ± 0.02	1.45 ± 0.01	1.37 ± 0.01	1.34 ± 0.02	1.23 ± 0.03
Protein efficiency^*∗∗*^	3.78 ± 0.10^a^	3.83 ± 0.12^a^	4.13 ± 0.10^a^	3.53 ± 0.15^b^	1.38 ± 0.17

Mean ± standard error (SE) of six rats. Superscript letters indicate a significant difference (Tukey's test, *p* < 0.05).

^*∗*^Food efficiency: body weight gain (g/day)/food consumption (g/day).

^*∗∗*^Protein efficiency: body weight gain (g/day)/protein consumption (g/day).

**Table 2:** Faecal and urine nitrogen content in rats fed a diet containing EW (unheated, soft-boiled, or boiled), milk whey protein, or soy protein.

	Unheated EW	Soft-boiled EW	Boiled EW	Milk whey protein	Soy protein (reference)
Faecal weight (dry g/5 days)	6.07 ± 0.10	6.15 ± 0.16	6.29 ± 0.10	6.27 ± 0.39	6.06 ± 0.24
Urine volume (mL/5 days)	55.1 ± 29.9	42.3 ± 6.9	39.0 ± 5.7	27.8 ± 4.3	14.5 ± 1.1
Faecal nitrogen (mg/5 days)	73.4 ± 3.1^a^	76.0 ± 4.2^a^	81.5 ± 1.8^b^	99.4 ± 2.4^c^	95.2 ± 2.4
Urine nitrogen (mg/5 days)	119 ± 21^a^	162 ± 24^a^	146 ± 20^a^	275 ± 26^b^	727 ± 39

Mean ± SE of six rats. Superscript letters indicate a significant difference (Tukey's test, *p* < 0.05).
